# Resource constrained neural network training

**DOI:** 10.1038/s41598-024-52356-1

**Published:** 2024-01-29

**Authors:** Mariusz Pietrołaj, Marek Blok

**Affiliations:** Faculty of Electronics, Telecommunications, and Informatics, Gdansk University of Technology, Gdańsk, Poland

**Keywords:** Computer science, Software, Computational science

## Abstract

Modern applications of neural-network-based AI solutions tend to move from datacenter backends to low-power edge devices. Environmental, computational, and power constraints are inevitable consequences of such a shift. Limiting the bit count of neural network parameters proved to be a valid technique for speeding up and increasing efficiency of the inference process. Hence, it is understandable that a similar approach is gaining momentum in the field of neural network training. In the face of growing complexity of neural network architectures, reducing resources required for preparation of new models would not only improve cost efficiency but also enable a variety of new AI applications on modern personal devices. In this work, we present a deep refinement of neural network parameters limitation with the use of the asymmetric exponent method. In addition to the previous research, we study new techniques of floating-point variables limitation, representation, and rounding. Moreover, by leveraging exponent offset, we present floating-point precision adjustments without an increase in variables’ bit count. The proposed method allowed us to train LeNet, AlexNet and ResNet-18 convolutional neural networks with a custom 8-bit floating-point representation achieving minimal or no results degradation in comparison to baseline 32-bit floating-point variables.

## Introduction

In recent years we saw a significant growth of neural network (NN) usage in various domains including computer security, business, agriculture, healthcare, finance, or military. The use cases focus on approximation of complex algorithms, computer vision, speech recognition, data classification, and many more^1^. Although novel applications of NN are limited only by researchers’ creativity, there are strict hardware requirements when it comes to both training and inference of NN models^2^. Most of the globally available general-purpose hardware leverages 32-bit single-precision IEEE 754 floating-point (FP32) format for calculations requiring a large dynamic range^3^. The problem lies behind FP32 computations that take a significant part in overall core energy consumption, including operations and moving operands between data memory and registers^4^. The majority of neural network designs heavily depend on a large number of FP32 multiplications resulting in high power and memory requirements. Hence, there is debate about deep learning with relation to growing energy consumption and its influence on carbon emission^5,6^.

Multiple effective methods of NN inference optimization have been already proposed. Various techniques such as pruning, quantization, or dynamic parameters limitation enable faster and more energy efficient inference, also on edge devices^7–10^. Hardware related research goes on par with algorithmic advancements. There are both experimental and production devices available in the form of neural network accelerators or co-processors such as DianNao, Intel MovidiusX, or Google TPU Edge^11–13^. Even though the aspect of inference has been well covered by multiple production-ready frameworks and hardware architectures, resource constrained NN training is still an open issue^14–16^.

Training of a modern, deep neural network requires significant computational resources and a large amount of input data. Therefore, powerful computational units need to be utilized to finish such a process in a reasonable time^2^. Graphical Processing Units (GPU) are commonly used for this purpose, but such an approach comes at the cost of the hardware and consumed power^3^. Limiting the time and resources required for NN training would allow for shortening time-to-product for many algorithms. Moreover, it would enable online training of specific models on edge devices without resource-expensive re-training and deployment. Adjusting the model on the device would allow limiting resource-consuming client–server communication and externally triggered model updates^17^. This is especially important in the case of privacy sensitive applications, where storing personal data outside of a device may not only undermine customers’ trust but also violate legal regulations^18^.

The continuously growing size of NN models and the high number of complex multiplications, required for both forward and backward passes, is the main root cause behind resource demanding NN training process^19^. Although due to a proper quantization, inference can often use fixed-point parameters to provide sufficient results, there are several studies showing that such an approach in the case of NN training might be difficult for variables below 12 bits^20–23^. Recent findings show that limiting the bit count of FP32 parameters tends to be the right path for improving power efficiency of modern NN^24,25^. Besides removing the least significant bits of the variables’ exponent and mantissa, researchers tend to propose mixed precision networks and sophisticated rounding techniques in order to avoid accuracy degradation^26–29^. In the result, reducing both memory and computational power required by multiplication operations can significantly increase the applicability of NN training to a broader number of devices. It has to be mentioned that major companies in the business as Nvidia or IBM already focus on low- and mixed-precision hardware for neural network training^30,31^. Additionally, there is growing interest in the hardware enabling calculations on flexible bit count parameters which is required for productization of many of the recently proposed algorithms^32–34^.

In our previous research we have shown that limiting floating-point parameters along with changing its bit-level representation allows for achieving accuracy close to FP32 baseline^35^. Based on this initial idea, the presented work provides an original contribution in terms of analysis of exponent values utilization during convolutional neural network training. Additionally, a new floating-point format has been proposed, including a custom approach to exponent range representation. A new method focusing on low-precision floating-point arithmetic for NN training has been presented combining techniques such as asymmetric exponent, stochastic rounding, and denormalization of low-precision variables. Moreover, extensive experiments on the proposed method’s impact on the selected NN architectures' training accuracy have been conducted, proving the method’s achievements. Our refined method shows that usage of limited floating-point value with asymmetric exponent, exponent offset, and stochastic rounding techniques enables efficient convolutional neural network training with a custom 8-bit floating-point.

This paper is organized in the following way, in the next section, related work and recent findings in terms of neural network training with limited precision are presented. Sections three and four give a detailed overview of limitation methods and experiments conducted during our research. In the fifth section, we present a summary of accuracy achieved for chosen convolutional networks along with a comprehensive results overview of the previously described papers. The ending sections combine the conclusions drawn from our work and future research directions.

## Related work

NN training optimization is still a vital subject of research as presented in our previous study^35^. This time, in order to further investigate the propositions from the recent work, we focus solely on findings published since 2020. The experiments reviewed in this section show intensified focus on NN optimization by precision limitation and modification of floating-point representation. There is a growing number of proposals leveraging mixed precision for limitation of inference resource requirements, combining in-training^36–39^ and post-training techniques^40^. More importantly, it opens additional research paths for power efficient neural network training.

In research towards energy-efficient neural network training Lee (2020)^14^ proposes a DNN training method called fine-grained mixed precision (FGMP). The technique is based on using both FP8 and FP16 in dynamically calculated ratio during the NN training in order to limit power requirements while maintaining the network’s accuracy. According to the author, the external memory accesses have been reduced by 38.9% for ResNet-18 training. In addition, a deep learning neural processing unit (LNPU) is proposed, allowing for doubling energy efficiency. In the case of ResNet-18, the method achieves FP16 levels of accuracy for both CIFAR10 and ImageNet datasets.

Along with the growing number of dynamic precision training algorithms, there is a visible demand for hardware architectures supporting such use cases. Precision-controlled memory system (PCM) proposed by Kim et al. (2020)^40^ focuses on reducing power requirements while training NN with limited parameters bit counts. Their work shows that it is possible to achieve FP32 accuracy of ResNet-20 on CIFAR100 with 34% lower energy consumption and 20% speed up in comparison to regular GPU architectures.

Another approach to mixed precision NN training is depicted by Rios et al. (2021)^41^. The method combines 16- and 32-bit arithmetic, where Brain Floating Point based half-precision stands for up to 96.4% of the computations. Experiments on AlexNet, Inception, and ResNet-50 showed accuracy results close to the FP32 baseline.

Fu et al. (2021)^42^ propose Cyclic Precision Training (CPT) which explores the idea of increasing variables’ bit count along training iterations. The authors state that the precision of NN parameters can be treated similarly to learning rate, its adjustment allows a network to generalize or converge depending on the bit count used. The method has been validated across multiple topologies such as ResNet, MoblieNet, LSTM, and Transformer achieving accuracies on par with FP32 implementations. A slightly different view on this matter is proposed by Yu et al. (2022)^43^. Their Learnable Dynamic Precision (LDP) framework uses additional layer-wise parameters for learning the optimal precision. The results show improvement in comparison to SBM^44^ or CPT^42^ techniques based on various ResNet models.

Park et al. (2022)^15^ present another approach to limited precision training with the use of 8-bit floating point with a shared exponent bias (FP8-SEB). The method also includes multiple-way fuse multiply–add (FMA) trees in hardware implementation. FP8-SEB consists of a tensor with FP8 values where 1 bit is assigned to sign, 4 to exponent, and 3 to mantissa. The exponent is biased differently for each tensor based on its dynamic range. According to the authors, the overhead of using separate biasing is negligible. Training results verified on ResNet-18 and ImageNet dataset achieve 69% accuracy. Moreover, the authors state that their hardware proposal requires 78.1 times lower energy than standard GPUs.

The architecture proposed by Junaid et al. (2022)^16^ leverages a mixed precision training approach, incorporating 32-, 24-, and 16-bits floating-point parameters along with the hardware accelerator engine. The solution includes a custom floating-point representation proposal and has been verified on a CNN with MNIST dataset achieving 93.32% accuracy versus 96% FP32 baseline. According to the authors, their mixed precision accelerator engine limits energy consumption by 3.91 times in comparison to FP32 architecture.

As presented in multiple cases^14,16,40–43^, novel methods focus on a static or dynamic mix of parameters with varying bit count. Although there are studies on hardware supporting such use cases, this approach provides an additional overhead on the design itself^33,45^. Our method also includes the ability of mixed precision application during NN training but does not enforce multiple bit count changes. In a similar fashion to FP8-SEB^15^, in our case, such functionality is achieved with the exponent offset method presented in the following chapter. Moreover, focusing on an optimal bit allocation to exponent and mantissa parts of a limited floating-point type is an important matter. In this work we investigate a full range of exponent and mantissa bit count combinations, including the previously mentioned variant with 4-bit exponent and 3-bit mantissa as its performance may vary per selected network architecture or training dataset.

## Limitation method

As already mentioned, in our research we have focused on refining the previously presented method of neural network training with asymmetric exponent^35^. The technique allowed us to train LeNet CNN without accuracy degradation on 12-bit floating point. The limitation assumed shortening FP32 exponent and mantissa to a given limited bit count. Instead of using a regular IEEE 754 exponent format, the asymmetric method assigns all bits to represent negative exponent values. In the case of all presented experiments, a general-purpose hardware has been utilized without any application of specific neural accelerators. Based on trainings conducted by the authors, even a configuration combining of Intel Core i7-4770, 32 GB 1600 MHz DDR3 RAM and GeForce GTX 1080 TI 11 GB is sufficient for results reproduction. Nevertheless, as most of available GPUs with CUDA capabilities should suffice, it is advised to use a more powerful hardware for NN training speed up. All calculations have been executed with software level limitation. All parameters and intermediate values were stored and calculated using 32-bit floating-point. The limitation to a given bit count was done after every calculation stage^21^.

In the current approach we provide a significant improvement of the previously proposed method, resulting in FP32 accuracy levels for LeNet, AlexNet, and ResNet-18 networks with 8-bit floating-point values. The new method includes:

- An additional offset of the asymmetric exponent.
- Introduction of stochastic rounding technique during the limitation process.
- Utilization of denormalized values for a limited floating-point type.

**Figure 1:**
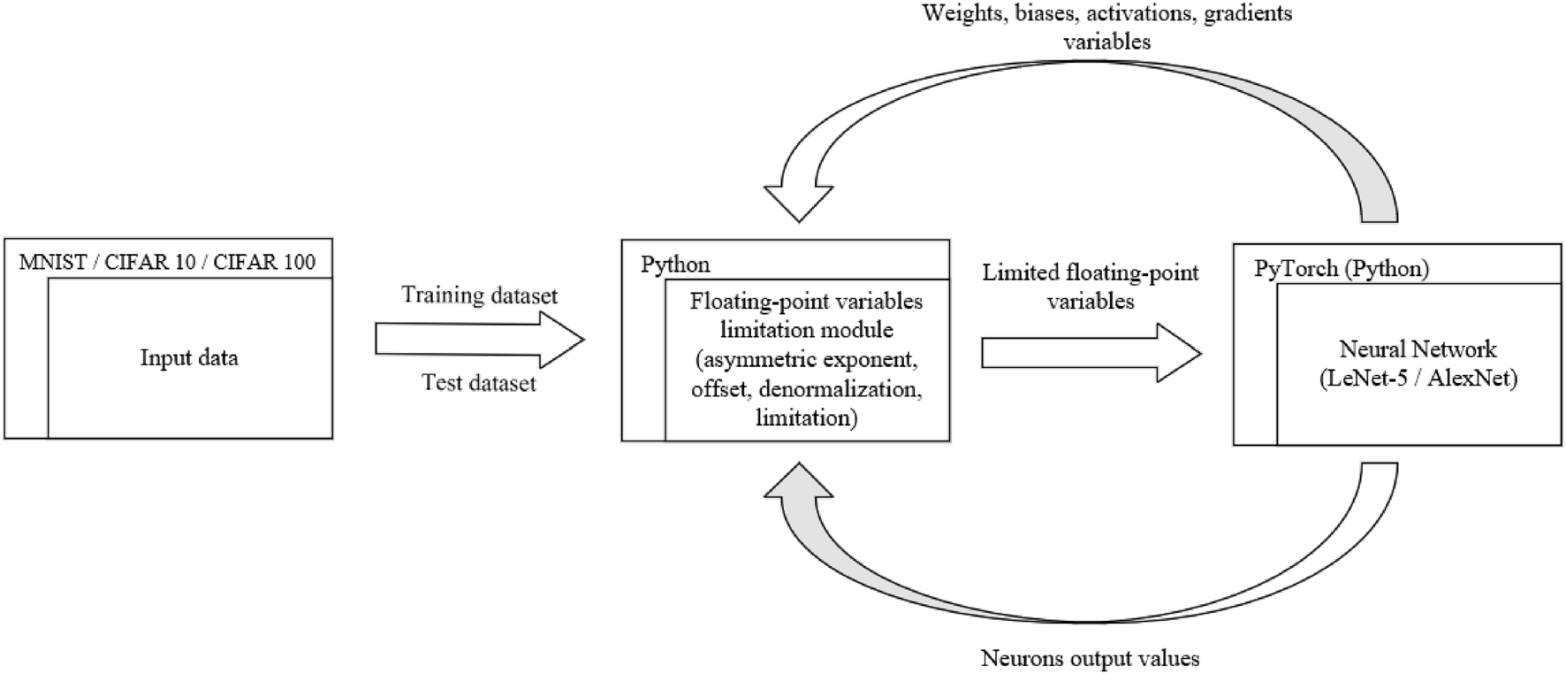
Overview of the proposed limitation method.

Figure 1 gives a general overview of the refined limitation method. The diagram presents the approach used in our experiments but does not include all available parameterization possibilities. The presented limitation method has been implemented with the use of Python 3.9 programming language and PyTorch 1.10 machine learning framework. Additionally, it leveraged cudatoolkit 10.2 and torchvision 0.11.2.

### Offset of the asymmetric exponent

**Figure 2:**
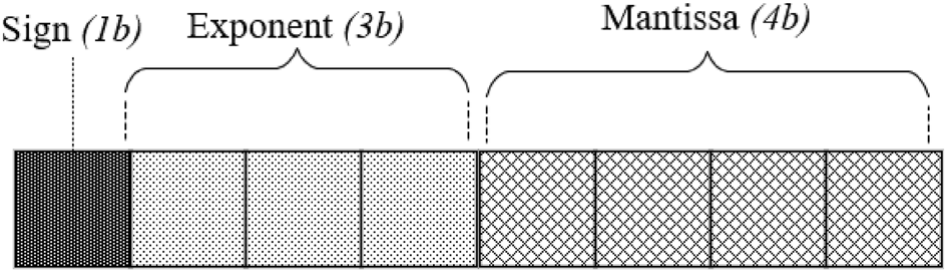
8-bit floating point value with 1-bit sign, 3-bit exponent, and 4-bit mantissa.

Based on our previous study, we introduced the asymmetric exponent method^35^. It was an answer for the low utilization of positive exponent values represented by a regular IEEE 754 representation during NN training. Such an approach allowed for limiting the bit count of FP32 without losing the commonly used dynamic range of floating-point parameters for a particular CNN. During our work, we discovered that selecting a specific range of negative values represented by the exponent improves the overall accuracy and training behavior of the network. Hence, applying an offset to an asymmetric exponent can be treated as an additional hyperparameter during the training process. Table 1 presents a comparison of 8-bit floating point variables with regular, asymmetric, and asymmetric with an offset exponent. The detailed format which assigns 1 bit to sign, 3 to exponent, and 4 to mantissa is presented in Fig. 2.

**Table 1:** Comparison of 3-bit exponent representations and their impact on 8-bit floating-point variable range.

Type	3-bit exponent value range	Full 8-bit variable range (4-bit mantissa)
Regular exponent	[−2, 3]	± [0.25, 15.5]
Regular exponent (no bits reserved for special values)	[−3, 4]	± [0.125, 31.0]
Asymmetric exponent	[−7, 0]	± [0.0078125, 1.9375]
Asymmetric exponent with offset set to 2	[−9, −2]	± [0.001953125, 0.484375]

### Introduction of stochastic rounding

A variety of new NN training studies include rounding techniques in their implementation. One of the commonly used methods is stochastic rounding which can be summarized by the following equation.

1 $$\begin{array}{c}r\left(x\right)=\lfloor x\rfloor+p, where\, p=\left\{\begin{array}{ll}0& with\; probability: 1 - \left(x -\lfloor x\rfloor\right)\\ 1& with\; probability: x-\lfloor x\rfloor\end{array}\right. \\ and\; x\; is\; in\; rational\; numbers \left(Q\right)\end{array}$$

Alternatively, the following version of Eq. (1) can be considered

2 $$\begin{array}{c}r\left(x\right)=\lfloor x+u\rfloor,\; where\; u\in \left[{0,1}\right) with\, uniform\, distribution \end{array}$$

which emphasize HW design efficiency improvement relying on the possibility of using random bit stream generator for generation of binary representation of stochastic parameter $u$.

In general, this rounding technique maps a number to the next smaller or larger value based on its distance between them. The smaller the distance, the higher probability of rounding to a particular value. The expected error of the stochastic rounding is zero, hence it allows to statistically preserve information about values in the limited NN^46^. The positive influence of stochastic rounding on NN accuracy has been confirmed by multiple experiments in case of limitation to both fixed-point and floating-point formats^20,47^. It is worth highlighting that besides mentioned benefits, this technique may introduce an additional overhead on the limitation method itself including NN acceleration hardware designs.

### Utilization of denormalized values for a limited floating-point type

In comparison to our previous work an extended approach to FP32 limitation is applied. Similarly as in the case of the original IEEE-754 standard, the denormalization range is established for a proposed limited floating-point type. Such approach provides improved utilization of available values range by additional representation of numbers that are close to zero. This mechanism is provided at the expense of the significant mantissa’s bits including interpretation of its hidden bit as 0. The denormalization feature for limited floating-point type can be simulated at the software level by simple bit shift operations. By usage of right bit shift, the targeted value is divided by 2 as long as there is at least one significant bit left in the mantissa’s representation. Such a limited value can then again be translated to a normalized floating-point representation with left bit shift operations and a proper exponent’s value adjustments.

Although the presented work mainly focuses on 8-bit parameters, such variables should not be treated as the final target of floating-point limitation. Nevertheless, selecting an appropriate minimal bit count or floating-point type representation might be difficult in the case of a variety of available NN topologies. Taking this into consideration, the proposed method and its experimentation framework treat these limitation factors as a part of the model’s hyper-parameterization. The assumption is that these characteristics can be dynamically adjusted during succeeding training epochs, which is often the case in recent NN limitation studies^42,43^. An additional advantage of this proposal is the possible parameterization of the exponent offset, which can also be dynamically modified during the training. Such an approach allows for changing the dynamic range of a limited floating-point parameter without affecting its bit count definition. The main advantage behind fixed bit count in the limitation to a targeted format is a simplification of future requirements for software algorithms or hardware designs.

## Conducted experiments

The proposed limitation method has been verified on three well-known neural network architectures, LeNet, AlexNet, and ResNet. The selection of these topologies was dictated by three main factors. The first one is easy reproducibility and comparison of our experiments’ results due to the popularity of these neural networks. The next one is the less demanding computational complexity of such CNN models, in comparison to much deeper networks, which allows for robust simulation-based experiments on general purpose hardware across all possible floating-point bit count variants in a reasonable time. Finally, many of new neural networks that aim for specific use cases are not as deep in their design, especially if power efficiency or embedding the model inside the chip’s memory is one of the goals. The selection of training data has been done based on similar arguments. The training leverages three publicly available datasets used for image classification tasks, MNIST, CIFAR10, and CIFAR100. These datasets provide a solid base for a benchmark comparison between other proposals of precision limitation algorithms for neural networks. Additionally, their moderate size and complexity allows for robust experimentation through a broad scope of floating-point format variants. Nevertheless, it is important to remark that the application of the proposed method is not limited to vision data or the selected datasets.

Table 2 presents hyper-parameters used during the conducted trainings along with their values in order to enable easy reproducibility of the presented results. It is important to highlight that hyper-parameters related settings remained identical for limited and regular training scenarios.

**Table 2:** Hyper-parameters used during trainings (presented per neural network architecture).

	LeNet	AlexNet	ResNet-18
Optimizer	Stochastic Gradient Descent	Adam	Stochastic Gradient Descent
Learning rate	0.01	0.0001	0.1
Batch size	64	128	128
Loss function	Cross entropy	Cross entropy	Cross entropy
Momentum	Not applicable	Not applicable	0.9
Weight decay	Not applicable	Not applicable	5e−4
Learning rate scheduler milestones	Not applicable	Not applicable	60, 120, 160
Learning rate scheduler gamma	Not applicable	Not applicable	0.2

Although the MNIST dataset remained unchanged, additional augmentations have been applied to train parts of CIFAR datasets. Table 3 gives a summary of transformations applied to both CIFAR10 and CIFAR100 across all conducted trainings along with required parameterization. All mentioned operations leverage implementation provided by torchvision package.

**Table 3:** Transformations applied to CIFAR10 and CIFAR100 datasets.

	CIFAR10	CIFAR100
Random Horizontal Flip	Probability: 0.5	Probability: 0.5
Random Crop	Height: 32, width: 4	size: 32, padding: 4
Random Rotation	Not applied	Degrees: 15
Normalization	Means (per channel): [0.485, 0.456, 0.406] Standard deviations (per channel): [0.229, 0.224, 0.225]	Means (per channel): [0.5070751592371323, 0.48654887331495095, 0.4409178433670343] Standard deviations (per channel): [0.2673342858792401, 0.2564384629170883, 0.27615047132568404]

**Figure 3:**
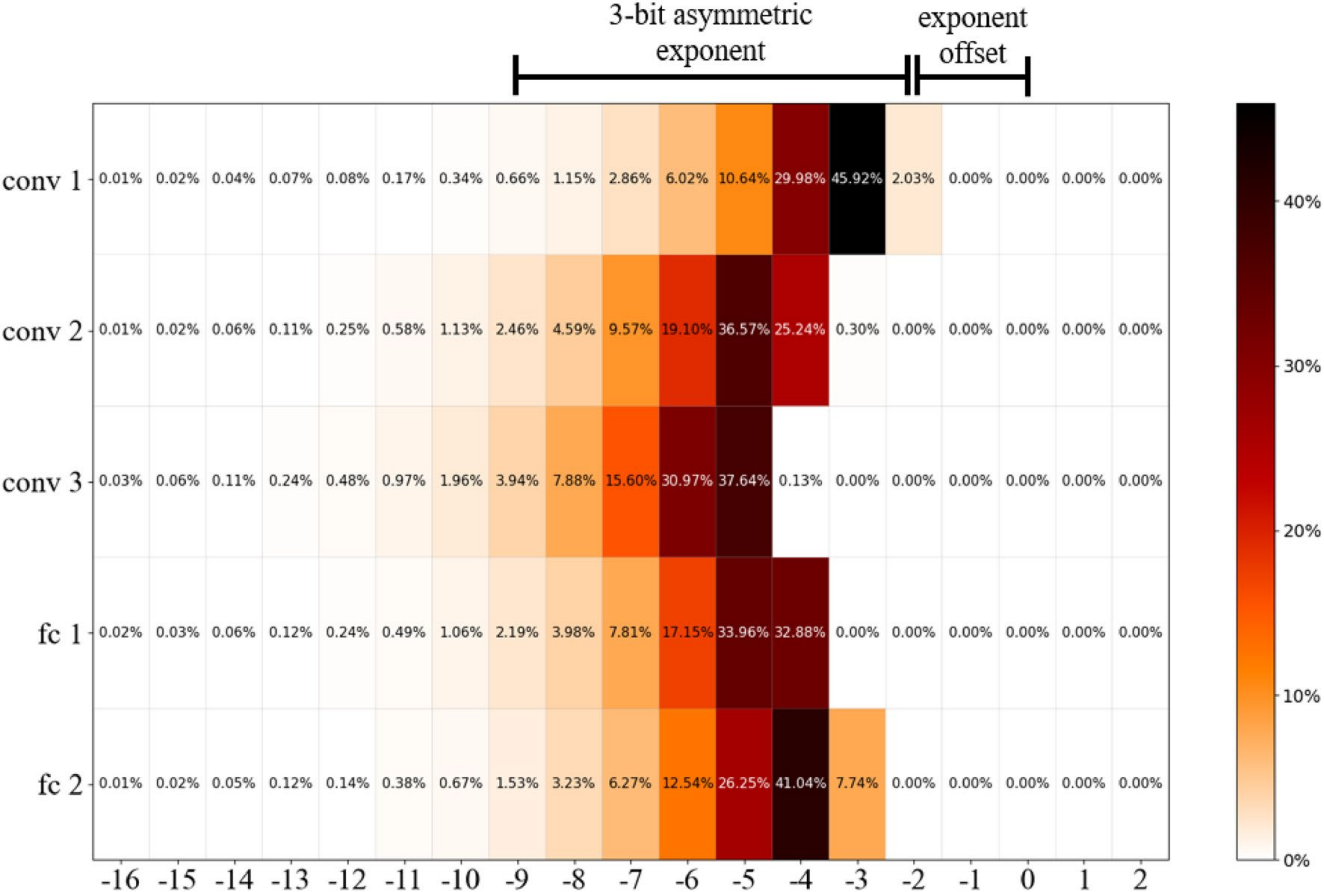
LeNet exponent values utilization for NN weights per layer (the darker the color the higher the utilization).

Asymmetric exponent values stored in limited floating point are additionally increased by the exponent offset that has been determined based on exponent utilization during FP32 baseline training. In the case of LeNet we have verified that across all network layers, exponent values utilized for the majority of weight parameters are located in the range from −9 to −2 (Fig. 3). Nevertheless, some small utilization can be also observed for lower exponent values, especially in the case of the range from −11 to −10 but as we will demonstrate their omission has no significant impact on the training accuracy. Based on this analysis for LeNet the selected limitation method includes an asymmetric exponent with offset set to 2, which covers most of the required exponent values. This approach has been applied to all weights, biases, and gradients of the neural network.

**Figure 4:**
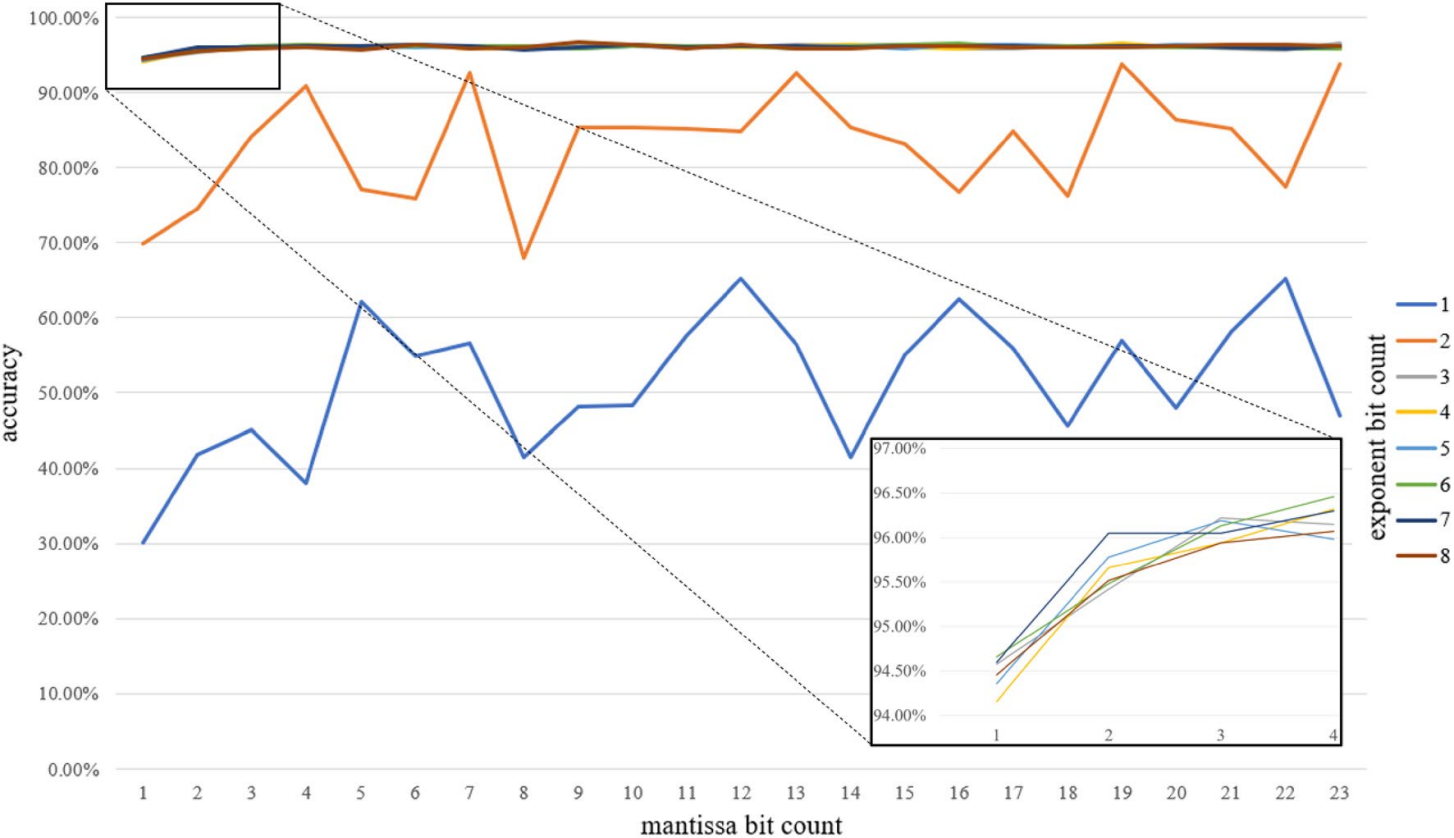
LeNet cross-validation accuracy with different exponent and mantissa sizes.

The training results have been verified across all bit count combinations available in 32-bit floating point, from 3 to 32 bit. Figure 4 presents a summary of LeNet accuracy over 10 epochs for the full range of exponent and mantissa bit counts combinations. Our experiments show that 8 bit-floating point variables are sufficient to train LeNet across the same number of epochs with no accuracy degradation in comparison to FP32 parameters on MNIST dataset.

Based on Fig. 4 we can see that, similarly to our previous research, 1- or 2-bit exponents are not sufficient to train a LeNet network without significant accuracy decrease. In the case of such highly limited exponents, the accuracy fluctuates even with continuously increasing mantissa bit counts. The accuracy starts to rapidly improve starting from 3-bit exponent and mantissa as low as 1-bit giving satisfactory 94.58% accuracy for a 5-bit floating-point. Even better results are observed for targeted 8-bit parameters allowing the network to achieve the FP32 baseline (Table 4) across 10 epochs. The 8-bit floating-point variant with 4-bit exponent and 3-bit mantissa achieves 95.98% versus 96.18% on FP32. Even better result of 96.15% can be achieved with 3-bit exponent and 4-bit mantissa. This also gives above 20 percentage points improvement in comparison to our previous result of 75.89%. It is worth mentioning that starting from this point, an additional increase of exponent and mantissa bit counts is not followed with an improvement of training accuracy which is presented by flat accuracy results lines for all exponents above 2 bits.

**Table 4:** Neural networks 8-bit floating-point accuracy across different variants of exponent and mantissa sizes.

8-bit floating-point variant			LeNet	AlexNet		ResNet-18	
Sign bit count	Exponent bit count	Mantissa bit count	MNIST	CIFAR10	CIFAR100	CIFAR10	CIFAR100
1	1	6	54.84%	22.23%	1.98%	8.02%	0.91%
1	2	5	77.81%	62.93%	1.46%	9.97%	1.02%
1	3	4	**96.15%**	72.94%	38.59%	**76.48%**	1.17%
1	4	3	95.98%	**74.50%**	**38.69%**	76.01%	40.21%
1	5	2	95.78%	71.10%	36.02%	62.85%	**42.62%**
1	6	1	94.66%	66.11%	30.00%	63.39%	39.68%
32-bit baseline			96.81%	74.39%	38.93%	77.08%	39.54%

Significant values are in bold.

**Figure 5:**
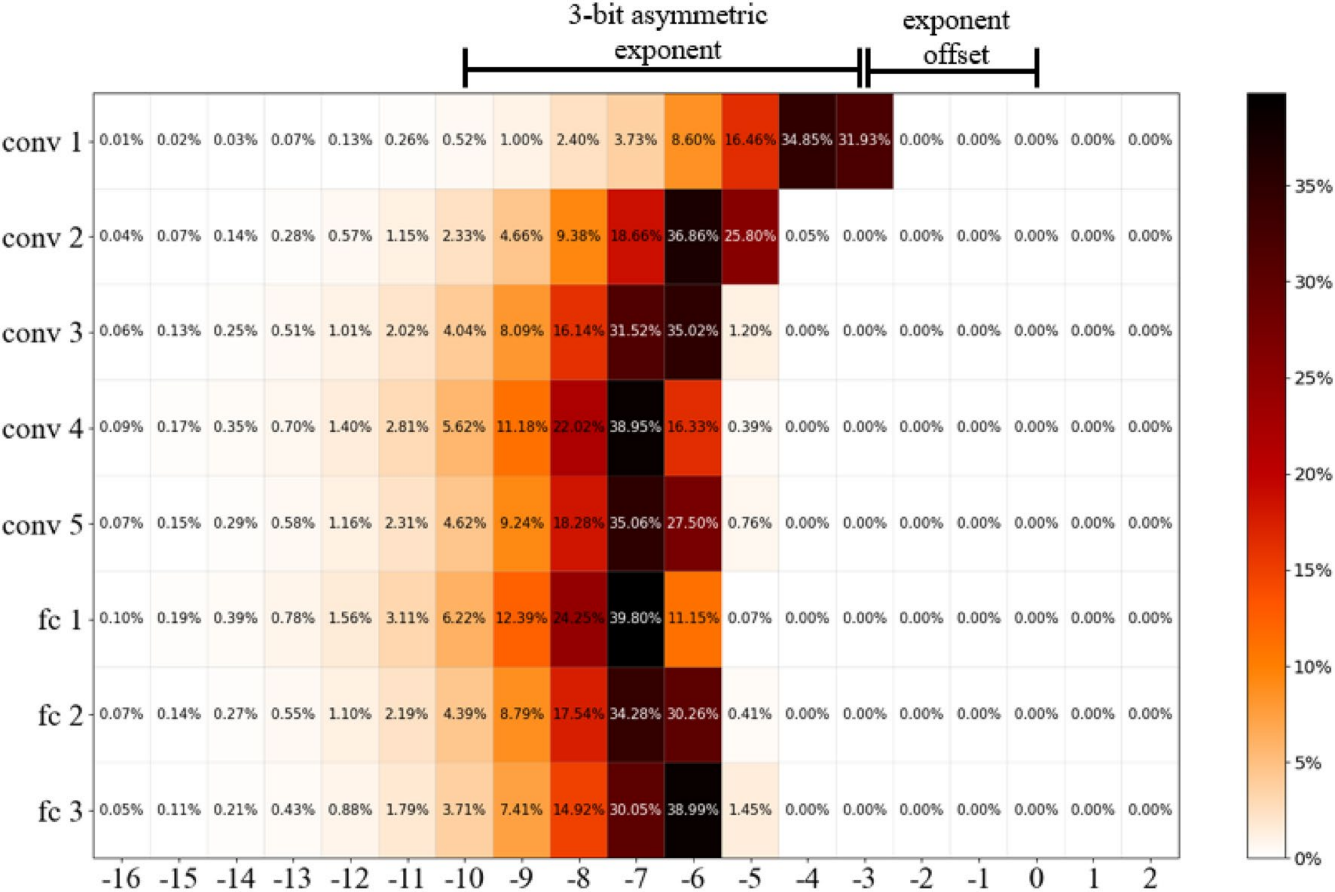
AlexNet (CIFAR10) exponent values utilization for NN weights per layer (the darker the color the higher the utilization).

Similar experiments have been conducted for AlexNet with CIFAR10 and CIFAR100 datasets. The only parameterization difference applied in the case of this network was the increase of the exponent offset by one. The reason behind this change comes from different exponent utilization derived from the FP32 training. Figure 5 gives an example of such analysis results based on exponent values utilization for AlexNet weights with CIFAR10 dataset. The selected asymmetric exponent’s range from −10 to −3 covers most exponent values used for weights during the training. The utilization of exponent values below −10 can be also observed, but they represent only a small percentage of parameters used per each layer. It is also worth remarking that the wider the range of exponent values is used, the more exponent bits will be required to maintain accuracy of the network, hence bigger topologies may require more bits for exponent representation.

**Figure 6:**
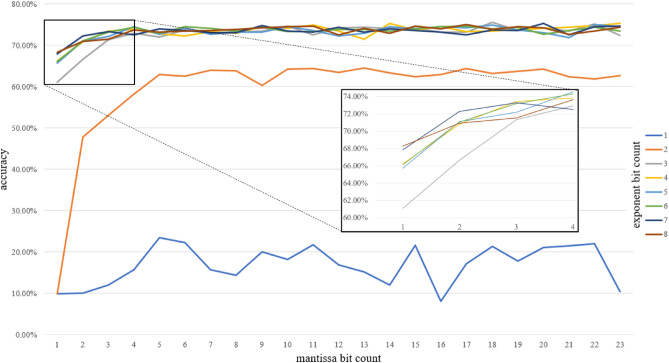
AlexNet cross-validation accuracy with different exponent and mantissa sizes (CIFAR10).

The CIFAR10 cross-validation results depicted in Fig. 6 show that the proposed technique allows to train a deeper convolutional network such as AlexNet with no accuracy degradation on 8-bit floating point. Same as with the LeNet, the 2-bit exponent is not enough to train the network. It can be observed that for AlexNet the results convergence is achieved for slightly higher bit counts. The 5-bit floating-point with 3-bit exponent and 1-bit mantissa is enough to achieve a tolerable result of 61.09%. Starting from a 3-bit exponent and 3-bit mantissa, the accuracy finally tends to follow the FP32 baseline of 74.39% (Table 4), although it is vivid that results fluctuation over increased bit counts is higher than in case of LeNet. Targeted 8-bit floating-point scenario with 4-bit exponent and 3-bit mantissa slightly outperforms the FP32 baseline with 74.5% accuracy over 10 epochs training.

**Figure 7:**
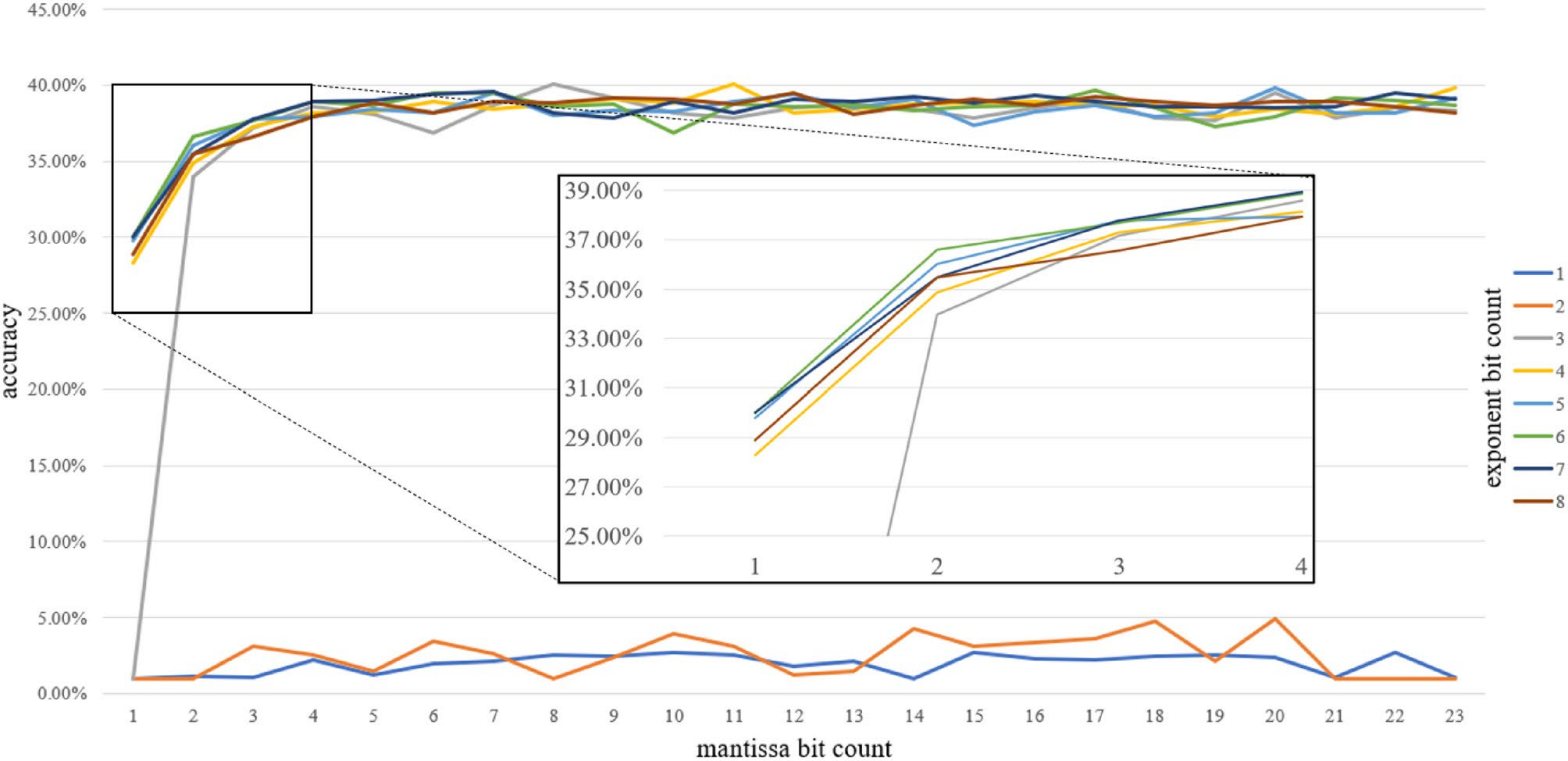
AlexNet cross-validation accuracy with different exponent and mantissa sizes (CIFAR100).

The next cross-validation experiment involved AlexNet with CIFAR100 dataset. The results of this verification are presented in Fig. 7. Same as with CIFAR10 the proposed method allows for training the network on 8-bit floating-point with no significant degradation in comparison to the FP32 38.93% baseline (Table 4). The limited network achieved 38.69% accuracy. In both scenarios, the networks have been trained over 10 epochs. Interestingly, the more complicated classification task stated in this experiment resulted in poor results for 5-bit floating-point. In case of such a limitation, it was not possible to train the network. First tolerable results are achieved for 6-bit floating-point with 33.98% accuracy.

Finally, the same framework parametrization as with AlexNet has been applied to ResNet-18. Although the network was unable to converge with as low as 5-bit floating-point variables, satisfactory results have been observed for 8-bit floating-point on CIFAR10. The baseline 32-bit accuracy of 77.08% (Table 4) was not matched by the limitation framework for 10 epochs giving 76.01% 8-bit counterpart with 4-bit exponent. However, the result can be improved to 76.49% by using 3-bit exponent and 4-bit mantissa type. The limited network was able to perform on similar accuracy over a standard training path of 200 epochs. In such a case the 4-bit exponent results gave a small advantage for 32-bit variables with accuracy of 94.99% vs 94.58%. Similar scenario has been observed for the CIFAR100 dataset. Accuracy on 10 epochs achieved 39.54% for 32-bit and 40.21% for 8-bit variables with a small advantage for limited network. In the case of 200 epochs, the limited network achieved satisfactory 8-bit accuracy of 74.25% in comparison to the 32-bit baseline of 75.08%.

**Figure 8:**
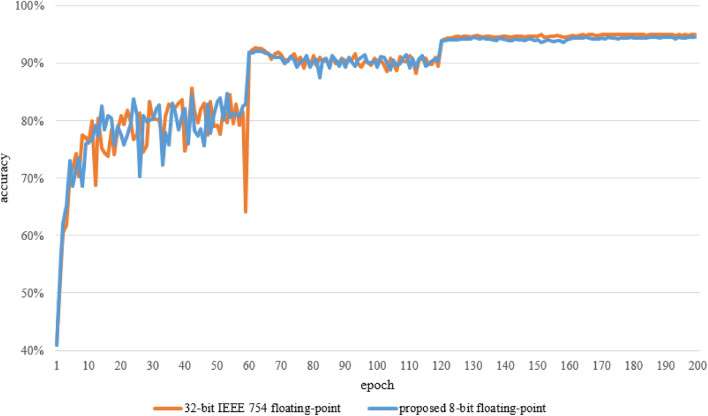
Comparison of ResNet-18 (CIFAR10) 32-bit IEEE-754 and proposed 8-bit floating point trainings convergence.

The training convergence is an especially important aspect when it comes to NN training with limited precision. The longer utilization of a training device may hinder expected power and memory savings. Hence, it is crucial that the proposed method does not negatively affect the time of the training convergence in comparison to regular 32-bit trainings. Figure 8 gives an example of network convergence between the proposed 8-bit limitation method and IEEE754 32-bit floating-point. The chart is based on the results achieved for ResNet-18 with CIFAR10 dataset. Additional data regarding network convergence for different epoch checkpoints can be found in the results section in Table 5.

**Table 5:** Summary of related study papers results including the proposed NN limitation method.

Paper	Variable type	Technique	Dataset	Topology	Baseline accuracy	Accuracy after limitation
Lee (2020) ^14^	Mix of: 16-bit FP 8-bit FP	Fine-Grained Mixed Precision	CIFAR10	ResNet-18	72.48% (16-bit FP)	72.45% (up to 94% of 8-bit FP)
			ImageNet		68.25% (16-bit FP)	69.11% (up to 90% of 8-bit FP)
Kim et al. (2020) ^40^ Subset of results presented	Mix of: 7-bit FP 9-bit FP	Precision-controlled memory system (PCM)	CIFAR10	ResNet-200	69% (16-bit FP)	~ 69% (9-bit FP)
Rios et al. (2021) ^41^	Mix of: 32-bit FP 16-bit Brain FP	Mixed precision training	ImageNet	AlexNet	60.79% (32-bit FP)	60.32% (BF16FMA 94.60%)
				Inception	74.01% (32-bit FP)	72.80% (BF16FMA 95.55%)
				ResNet-50	75.69% (32-bit FP)	92.70% (BF16FMA 96.40%)
Fu et al. (2021) ^42^ Subset of results presented	Dynamic range: From 2-bit FP to 32-bit FP	Cycling Precision Training (CPT) Last two stages trained with full precision	CIFAR10	ResNet-74	91.15% (SBM 6 bit)	92.4% (CPT 3-t o 6-bit, grad 6 bit)
				MobileNetV2	91.56% (SBM 6 bit)	91.81% (CPT 4- to 6-bit, grad 6 bit)
			CIFAR100	ResNet-74	70.31% (SBM 6 bit)	70.83% (CPT 3- to 6-bit, grad 6 bit)
				MobileNetV2	72.31% (SBM 6 bit)	73.18% (CPT 4– to 6-bit, grad 6 bit)
			ImageNet	ResNet-18	69.76% (32-bit FP)	70.67% (CPT: 8- to 32-bit)
Park et al. (2021) ^15^	8-bit FP	Floating point with shared exponent bias multiple-way fuse multiply–add trees	ImageNet	ResNet-18	Not defined	69% (8-bit FP + SEB)
Junaid et al. (2022) ^16^	Mix of: 32-bit FP 24-bit FP 16-bit FP	Mixed precision training	MNIST	Custom CNN	96% (32-bit FP)	93.32%
Yu et al. (2022) ^43^ Subset of results presented	Dynamic range: From 3-bit FP to 16-bit FP	Learnable Dynamic Precision (LDP)	CIFAR10	ResNet-18	91.86% (SBM 8 bit)	92.08% (LDP 3- to 8- bit, grad 8 bit)
			CIFAR100		67.24% (SBM 8 bit)	67.88% (LDP 3- to 8- bit, grad 8 bit)
			ImageNet		69.60% (SBM 8 bit)	69.62% (LDP 4- to 8- bit, grad 8 bit)
Our previous proposal. (Pietrołaj and Blok 2022) ^35^	8-bit FP 12-bit FP 14-bit FP	Asymmetric exponent No additional rounding	MNIST	LeNet	96.04%	75.89% (8-bit FP) 95.01% (12-bit FP) 97.13% (14-bit FP)
Current proposal	8-bit FP (4-bit exponent and 3-bit mantissa)	Asymmetric exponent Exponent offset Stochastic rounding	MNIST	LeNet	96.18% (10 epochs) 98.35% (30 epochs)	8-bit FP: 95.98% (10 epochs) 98.38% (30 epochs)
			CIFAR10	AlexNet	74.39% (10 epochs) 79.53% (30 epochs)	8-bit FP: 74.5% (10 epochs) 80.06% (30 epochs)
				ResNet-18	77.08% (10 epochs) 83.41 (30 epochs) 94.99% (200 epochs)	8-bit FP: 76.01% (10 epochs) 82.22% (30 epochs) 94.58% (200 epochs)
			CIFAR100	AlexNet	38.93% (10 epochs) 51.82% (30 epochs)	8-bit FP: 38.69% (10 epochs) 51.91% (30 epochs)
				ResNet-18	39.54% (10 epochs) 51.96% (30 epochs) 75.08% (200 epochs)	8-bit FP: 40.21% (10 epochs) 55.16% (30 epochs) 74.25% (200 epochs)

As presented in Fig. 8 the accuracy of the proposed method with 8-bit floating point closely follows the one achieved for the 32-bit. It can be observed that the stabilization of accuracy occurs at similar training stages in both cases around the 120th epoch. The initial training, up to 60th epoch, shows a much higher fluctuation of results but it remains in similar boundaries for both 32-bit and 8-bit scenarios. The steep changes observed in the chart can be attributed to learning rate scheduler’s milestones which were set to 60, 120, and 160 epochs. Interestingly, the IEEE-754 32-bit based training shows a much more significant reaction for the first learning rate milestone with a staggering decrease of accuracy below 15 percentage points. The 200th epoch’s accuracy difference does not exceed 0.5 percentage point.

The experiments presented in this section confirm that the proposed method allows for training convolutional neural networks with 8-bit or even lower floating-point parameters. In the case of a variety of network topologies and available datasets, the method’s hyper-parameterization is a crucial way for achieving satisfactory results. The presented technique aims to establish a consistent precision limitation method for neural network training with low bit count variables. The method’s applicability to other NN topologies may differ per chosen architecture and its size. A similar case should be considered in the case of datasets with different size, shape, and complexity. Hence, it is crucial to leverage the mechanisms provided by the proposed method as hyper-parameterization during the training stage. This includes the exponent bit count, its asymmetric representation and offset. Although presented experiments, leveraged constant values of the mentioned parameters during a single training, they can be dynamically adjusted to specific parameter’s types, layers or even training epochs. Such an approach provides a wide range of method’s enhancements including mixed-precision training procedures for larger neural topologies. The authors state that such a dynamic approach to modification of the proposed technique during training and its additional calibration gives a big margin for further improvements of the presented results.

## Results

As shown in the experiments section, the refined method of parameters limitation allowed for training CNNs without significant accuracy degradation for 8-bit floating-point parameters. Additionally, it was possible to train LeNet and AlexNet networks with MNIST and CIFAR10 datasets with as low as 5-bit floating-point. Our results show that proper selection of a bit count split between exponent and mantissa parts of floating-point type has a significant impact on final network’s accuracy. As previously mentioned, it can be observed that commonly used 4-bit exponent and 3-bit mantissa floating-point type is not always the optimal solution for limited precision network training and its performance may vary depending on chosen topology or dataset. Table 4 present the accuracy achieved for investigated networks with multiple variants of 8-bit floating-point bits distribution over 10 epochs. In each case, besides 32-bit baseline, the previously presented limitation method has been applied.

Table 5 gives a detailed overview of techniques presented in the related work section in comparison to the proposed NN limitation method. The summary gives a description of each solution along with datasets and topologies used during the evaluation phase. Moreover, the type of selected variables is highlighted as one of the major factors in NN limitation. To ensure that the comparison was unbiased stop-loss mechanism was not applied. Results were presented for 10 and 30 epochs variants to show that no significant degradation occurs along with the later stages of CNNs training. In the case of ResNet-18 additional case of 200 epochs is presented to allow full convergence of the tested topology. Unfortunately, direct comparison of the methods is difficult due to different training hyper-parameters, topologies, datasets, or even numbers of epochs used during training. In many cases such information is partially missing. Hence, the authors decided to present the accuracy results for each experiment in comparison to the baseline provided in the specific papers. In combination with various variable types, such an approach gives a general overview on the performance of the presented works.

**Figure 9:**
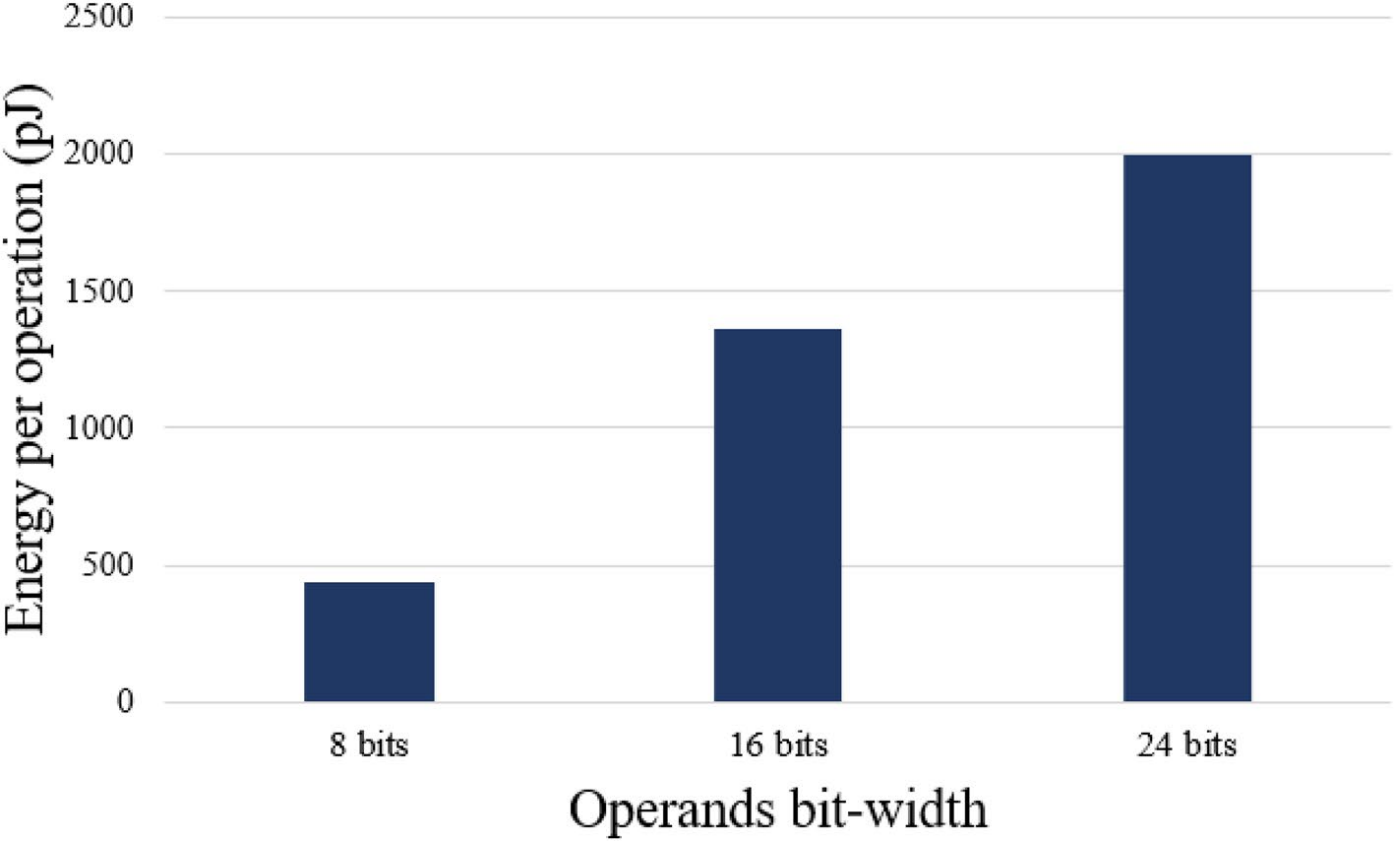
Performance of the digital multiplier across selected bit-widths^48^.

The proposed limitation method implies substantial resource savings. It is important to remark that it does not enforce additional floating-point operations as both neural network topology architecture and number of training epochs remain unchanged. Taking this into consideration, shortening regular FP32 to 8-bit representation requires up to 4 times less storage capacity and runtime memory. In addition, using 8-bit floating-point multiplications may reduce power consumption to less than a third of a regular 32-bit based unit^48^. Such savings are especially important in the case of low power edge devices where both power and memory consumption are the main constraints. Although power measurements are highly hardware dependent and are difficult to be precisely calculated in the simulated environment, Fig. 9 gives an example of energy required per floating-point operation across different variable bit-widths based on the research of Tong et al. (2000)^48^.

Based on the presented research, it can be stated that there is a clear correlation between the operands bit-width and energy consumed by a single floating-point operation. This is why limiting the bit count of floating-point variables, which is the root of our method, can be treated as one of efficient techniques of energy savings and the reduction of computational complexity.

## Future work

Taking into consideration a variety of neural network designs and hardware accelerators for flexible floating-point bit counts, moving the presented method from general purpose hardware simulation to custom designs is an obvious continuation of this research. Such an approach would not only allow to thoroughly validate the presented method, but also precisely measure both power and latency savings while using a limited NN model.

Automatic parameterization of the proposed method is another focus of the presented research. The authors work on a profiler implementation that would monitor regular FP32 training and gather statistics about exponent utilization for a selected NN. Based on such information the mechanism could propose the optimal per-layer or per-epoch parameterization for the limitation module. This feature should also allow for much more robust generalization of the presented limitation method across various neural network architectures and datasets.

The efficient rounding hardware implementation is another aspect of the research that is worth pursuing. Although stochastic rounding is an effective and well tested method, additional effort should be put into finding more power and hardware friendly techniques that can be easily introduced to low power devices.

## Conclusion

Training neural network models on low-power edge devices is mainly constrained by limited memory and power resources. Turning towards limited precision floating-point calculations creates a promising area for low-resource NN training. This paper touches on this issue by proposing an effective method of training convolutional neural networks as LeNet, AlexNet, and ResNet-18 with limited floating-point precision on MNIST and CIFAR datasets. A deeply refined asymmetric exponent method is presented with improvements like exponent offset, denormalization utilization, and stochastic rounding. The limited CNNs achieve on par results with the 32-bit floating-point baseline for proposed 8-bit floating-point parameters. Such an approach would allow for up to 4 times memory savings and potentially above 60% power consumption reduction with custom designed hardware.

## Data Availability

The datasets used for experiments are publicly available. MNIST: http://yann.lecun.com/exdb/mnist/. CIFAR10 and CIFAR 100: https://www.cs.toronto.edu/~kriz/cifar.html.
